# Learning to Identify Near-Acuity Letters, either with or without Flankers, Results in Improved Letter Size and Spacing Limits in Adults with Amblyopia

**DOI:** 10.1371/journal.pone.0035829

**Published:** 2012-04-30

**Authors:** Susana T. L. Chung, Roger W. Li, Dennis M. Levi

**Affiliations:** 1 School of Optometry, University of California, Berkeley, California, United States of America; 2 Helen Wills Neuroscience Institute, University of California, Berkeley, California, United States of America; CNRS - Université Claude Bernard Lyon 1, France

## Abstract

Amblyopia is a developmental abnormality that results in deficits for a wide range of visual tasks, most notably, the reduced ability to see fine details, the loss in contrast sensitivity especially for small objects and the difficulty in seeing objects in clutter (crowding). The primary goal of this study was to evaluate whether crowding can be ameliorated in adults with amblyopia through perceptual learning using a flanked letter identification task that was designed to reduce crowding, and if so, whether the improvements transfer to untrained visual functions: visual acuity, contrast sensitivity and the size of visual span (the amount of information obtained in one fixation). To evaluate whether the improvements following this training task were specific to training with flankers, we also trained another group of adult observers with amblyopia using a single letter identification task that was designed to improve letter contrast sensitivity, not crowding. Following 10,000 trials of training, both groups of observers showed improvements in the respective training task. The improvements generalized to improved visual acuity, letter contrast sensitivity, size of the visual span, and reduced crowding. The magnitude of the improvement for each of these measurements was similar in the two training groups. Perceptual learning regimens aimed at reducing crowding or improving letter contrast sensitivity are both effective in improving visual acuity, contrast sensitivity for near-acuity objects and reducing the crowding effect, and could be useful as a clinical treatment for amblyopia.

## Introduction

Amblyopia is a developmental abnormality that results from physiological alternations in the visual cortex and impairs form vision [1]. It is a leading cause of vision loss in infants and young children, affecting approximately 2–4% of the population. If detected and treated early, the vision loss in the amblyopic eye can be effectively reversed [2], [3]. Although individuals with amblyopia often retain good vision in the non-amblyopic eye, treatment to reverse the vision loss in the amblyopic eye is important for at least two reasons. First, to avoid the devastating impact in case there is an acquired loss of vision in the non-amblyopic eye later in life. Second, amblyopia is a consequence of abnormal binocularity [4]. The ultimate benchmark for “curing" amblyopia is the presence of functional binocularity, which requires similar levels of acuities in the two eyes [5], [6].

Conventionally, patching is the treatment of choice for amblyopia [7]–[9]. Disadvantages of patching include non-compliance from young children, the risk of further reducing binocularity and the loss of self-esteem [10]. Recently, perceptual learning has been proposed as an alternative, effective treatment to improve functional vision in amblyopia [2], [3]. A characteristic of perceptual learning is its specificity — that the improvement following perceptual learning is specific to the training task, although the degree of specificity has been shown to depend on the training conditions [11]–[13]. For perceptual learning to be an effective treatment for amblyopia, the improvements should be generalizable to include, at the minimum, good acuity, high contrast sensitivity and the ability to see objects in clutter.

A fundamental question in relation to applying perceptual learning to improve functional vision in amblyopia is whether the improvements are indeed related to the training task *per se*, or whether the improvements are the result of some more general improvements of visual processing, for instance, the ability of observers to extract the crucial information from the stimulus [14]–[17]. Astle, Webb & McGraw [18] compared the effects of training amblyopes on two types of tasks, targeted at fundamental visual deficits: contrast sensitivity tasks aimed at ameliorating the contrast sensitivity deficit, and acuity tasks, targeted at the acuity deficit. Their results suggest that training on the contrast sensitivity tasks produced substantial within-task learning and also generalized to measures of visual acuity. Training on a letter acuity task (varying letter size) also resulted in substantial, but somewhat smaller improvements in performance on the trained task, but did not generalize to contrast sensitivity.

An important limiting factor in amblyopic spatial vision is the ability to recognize objects in clutter. When the distance between adjacent objects is too small, object recognition is impaired — this is known as crowding, and it reflects a spacing limit. When objects are closer together than the spacing limit, crowding occurs. Many amblyopes, particularly strabismics, have substantial crowding in central vision [19]–[26]. Crowding has been shown to be a bottleneck on object recognition and reading in amblyopia [27], [28]. Therefore, reducing crowding is an important goal in ameliorating amblyopia. Previously, Chung [29] showed that following 6000 trials of repeated training to recognize the middle letter of sequences of three random letters (“*trigrams*") that were rendered in close spacing, the performance for recognizing the middle letter (the “crowded" letter) improved in the normal periphery. This improvement was accompanied by a reduction in the spacing limit, so that adjacent objects could be closer together and still be recognized.

The primary goal of this study was to evaluate whether it is possible to reduce crowding in adults with amblyopia through perceptual learning, using the same “flanked letter training" task as in Chung [29]. This task was specifically designed to reduce the spatial crowding effect in normal peripheral vision [29] so as to improve the ability to see objects in clutter, which is common in daily visual tasks. To evaluate whether the improvements following this training task were specific to training with flankers, we trained another group of amblyopic observers using a different letter identification task that did not involve flankers. This task, the “isolated letter training", was modified based on the grating contrast sensitivity training task used by Zhou et al [30] and our previous letter contrast sensitivity training studies [31], [32]. Reduced contrast sensitivity, particularly for fine details (high spatial frequency gratings or small letters), is a characteristic of amblyopia [33]–[35], therefore improving contrast sensitivity is also important in the treatment of amblyopia. To better relate to letter identification in daily life, we modified the task of detecting sine-wave gratings as in Zhou et al [30] to one that involved identifying near-acuity single letters. Astle et al [18] showed that training on contrast sensitivity tasks generalized to improvements in acuity for isolated targets, but here we also asked the question of whether the improvements would generalize to better performance in seeing objects in clutter (crowding). Our expectation was that the isolated letter training would not be effective in reducing crowding. However, as our results will show, both flanked and isolated letter training yielded similar magnitudes of improvements for the training tasks, as well as for a variety of untrained visual tasks (including crowding).

## Results

**Figure 1:**
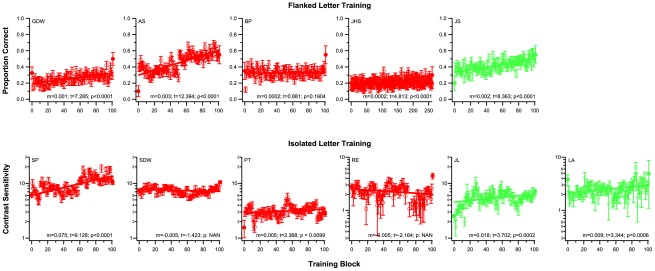
Training data for individual observers. The top row shows the data for the flanked letter training group while the bottom row shows the data for the isolated letter training group. Each observer (except for JHS who was trained for 26 sessions) participated in 10 sessions of training. In all panels, each unfilled symbol represents the performance for a block of 100 trials. Filled symbols on the leftmost and rightmost edge of each panel represent the data during pre-test and post-test. Error bars represent ±1 s.e.m. Linear regression function was used to fit each set of data. The slope of this function, if different from 0 (p-value given in each panel), implies significant improvement. The slopes of the linear function for observers SDW and RE in (b) were negative, thus the p-value for improvement was listed as “NAN". Note also the change in scale on the ordinate for observers RE and LA in (b). The slope of the regression line (*m*), the t-statistic in calculating the significance and the p-value are given in each panel.

We first established whether we could improve performance for identifying crowded letters in observers with amblyopia using the flanked letter training task, which was effective in reducing the spacing limit in the normal periphery [29]. Five observers (four with strabismus and one without, Table 1) participated in this training. The performance measurement during training was the proportion correct for identifying the middle letter of trigrams (see Materials and Methods for details). The stimulus array was, by design very crowded. Initially, on average, observers identified the middle letter correctly only 24% of the time. In contrast, they identified an unflanked letter of the same size ≈95% of the time, indicating a substantial effect of the flankers. Despite substantial individual differences which are typical for perceptual learning, all observers demonstrated improved identification accuracy over the course of training, from an average of 0.24 (proportion correct) in the first training block to 0.38 in the last training block (an average of approximately 60% improvement). Yet, these identification accuracies are still relatively low, and clearly reflect that the crowding task was challenging, even at the end of the training. Training data for individual observers are presented in the top row of Fig. 1. We quantified the improvements during training in three ways. First, we fit each observer's training data with a linear function, and examined whether the slope of the linear function was significantly different from a slope of zero by calculating the t-statistic of the slope (t = slope/standard error of the slope). The t-statistic and the degrees of freedom (number of data points – 2) were then used to determine the p-value. This method allows us to include all the data during training to determine if there was a significant improvement. Using this method, we determined that the slope for four of the five observers in the flanked letter training group was statistically different from zero. The one-in-five observer (20%) who did not show any improvement is similar to the percentage of “non-learners" reported in previous studies [17], [32], [36]. Second, based on the fitted linear function, we calculated the *expected* performance for the first and the last block of trials and quantified the improvement based on the ratio of these two calculated values. This ratio, averaged across observers, was 0.69±0.15 (95% CI). The third method we adopted to quantify the improvements was to calculate the ratio of the *empirical* performance between the first and the last block of trials, akin to comparing performance *“before"* and *“after"* training. While this method does not take into account all the training data, it is a standard way to compare improvements due to training especially when comparisons with untrained tasks are to be made (for a review of studies that used this method, refer to [2]). Averaged across observers, the ratio between the first and the last block of trials was 0.60±0.19 (95% CI). Regardless of whether the ratio between the first and the last block of training was based on the calculated values from the fitted linear function or from the empirical data, a ratio of 1, meaning that there was no change in performance between the first and the last block of trials, did not fall within the 95% confidence intervals. Therefore, we infer that the improvement was significant at α = 0.05.

**Table 1:** Visual characteristics of the 11 observers.

Observer	Gender	Age	Type	Eye	Visual Acuity	Refractive Errors	Eye Alignment
		(years)			(logMAR)		
***Flanked Letter Training***							
GDW	M	23	Strab	OD	20/32+1 (20/20+2)	+3.25	6Δ RET
				OS	20/12.5−1	+2.50	
BP	M	67	Strab	OD	20/32+2	−7.50	
				OS	20/400−2 (20/100−2)	−2.00/−2.25×005	10Δ LET
AS	F	32	Strab	OD	20/63−1 (20/50+2)	pl/−1.00×120	
				OS	20/16−1	−4.00	8–10Δ LET
JHS	F	53	Strab	OD	20/16+1	+1.25/−0.50×150	
				OS	20/125−2 (20/63)	+1.00/−0.50×160	>30Δ LXT
JS	F	26	Non-strab	OD	20/25−2 (20/25+2)	+1.00	4Δ EsoPhoria
			(Aniso)	OS	20/12−2	+0.25	
***Isolated Letter Training***							
SP	F	22	Strab	OD	20/80−2 (20/40−1)	+0.75/−1.50×090	10–12Δ RXT
				OS	20/12	−0.25	
SDW	F	46	Strab	OD	20/12.5−1	+2.00	6Δ RHyperT
				OS	20/40−1 (20/25−1)	+3.00/−0.75×095	25Δ LXT
PT	F	40	Strab	OD	20/12.5+1	pl	
				OS	20/32+2 (20/25+2)	+1.75/−0.50×005	>25Δ LET
RE	F	27	Strab	OD	20/40−1 (20/25−2)	−0.50/−3.75×150	20–25Δ RET
				OS	20/20−2	−2.00/−3.50×025	
JL	M	30	Non-strab	OD	20/16+1	−1.50/−0.25×160	4Δ EsoPhoria
			(Aniso)	OS	20/63+1 (20/50+2)	+0.75/−0.75×170	
LA	F	47	Non-strab	OD	20/50−2 (20/50−2)	+4.25/−4.00×072	
			(Aniso)	OS	20/16−2	+0.25/−1.00×115	

Acuities are measured using a Bailey-Lovie Chart. Values in parentheses represent single-letter acuities.

**Figure 2:**
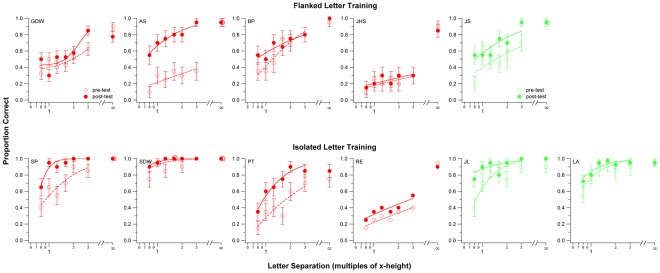
Proportion of correct responses in identifying flanked letters as a function of center-to-center letter separation in trigrams, for the task of measuring the spacing limit, is plotted for each individual observer. Letter separations are specified as multiples of the x-height. Unfilled symbols represent pre-test results and filled symbols represent post-test results. The smooth curve drawn through each data-set represents a cumulative-Gaussian function fitted to the data, from which we define the spacing limit as the letter separation that yields 0.52 on the cumulative function. The rightmost points (for a separation of ∞) represent performance for identifying single (unflanked) letters. The two data points are offset slightly to avoid clutter. Error bars represent ±1 s.e.m.

**Figure 3:**
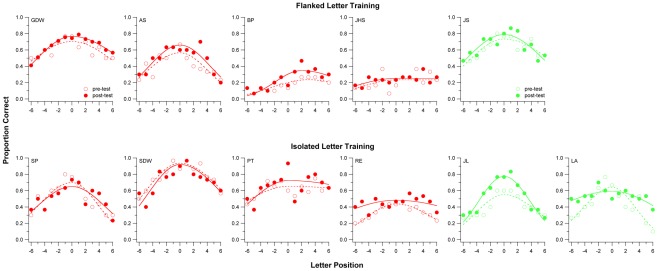
Proportion of correct responses in all three letters in trigrams, presented at different letter position left and right of fixation, for the task of assessing the visual span. Data are plotted for each individual observer. Unfilled symbols represent pre-test results and filled symbols represent post-test results. The smooth curve drawn through each data-set represents a split-Gaussian function fitted to the data. The size of the visual span, akin to the measurement of the area under the curve, was quantified by first converting each proportion-correct value (from the fitted curve) to bits of information transmitted, then summing up these values across all letter positions (values plotted in Fig. 4d).

**Figure 4:**
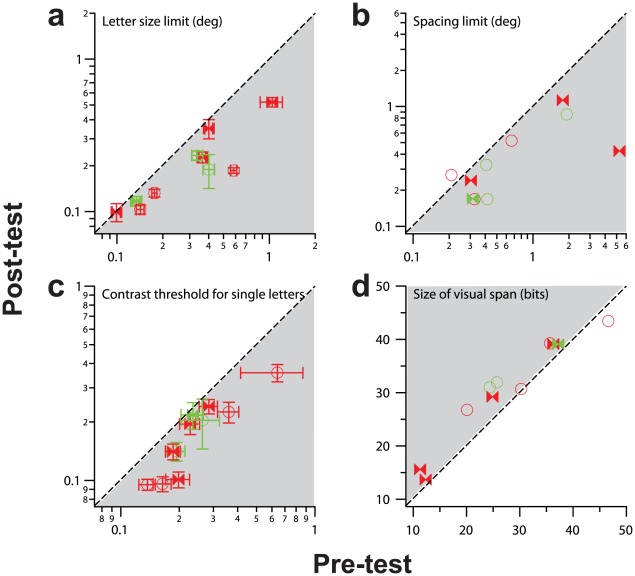
Comparisons of the post- and pre-test performance for four untrained visual tasks. **a.** The letter size limit (acuity) in degrees of visual angle. **b.** The letter spacing limit (defined as the letter separation that yielded 52% on each fitted function in Fig. 2), converted to degrees of visual angle by multiplying the estimate with letter size. **c.** Contrast threshold for identifying single letters. **d.** The size of the visual span in bits of information transmitted. In each panel, the dashed line represents the 1∶1 line and the light gray shaded region represents improvement. Each symbol represents data for one observer, with red representing strabismic amblyopes and green representing non-strabismic amblyopes. Filled bowtie symbols represent observers trained on the flanked letter task and unfilled circular symbols represent observers trained on the isolated letter task.

To determine whether the improvement following the flanked letter training transferred to other untrained visual tasks, we compared four measurements related to various aspects of identifying letters before and after training. These four measurements were: (1) the *size limit (visual acuity)*, the smallest letter size that was required for observers to identify single letters at 52% correct; (2) the *spacing limit*, the letter separation between adjacent letters such that the performance of identifying the middle letter of trigrams was 52% correct (Fig. 2), representing a measure of the distance over which crowding occurs; (3) the *contrast threshold* for identifying single letters; and (4) the *size of the visual span profile*, the amount of information of the letter stimuli that was transmitted in a fixation (Fig. 3). These four performance measures utilize similar, highly familiar stimuli (letters) and responses (letter identification), thus minimizing procedural learning. Fig. 4 summarizes these comparisons. In each panel, each symbol represents data from an individual observer (red – strabismic amblyopes; green – non-strabismic amblyopes; bowtie symbols – flanked letter training group; circular symbols – isolated letter training group, see later). For panels a–c, data points plotted below the diagonal 1∶1 line and in the shaded region represent improvement (values being smaller for post-test than for pre-test); whereas for panel d (size of the visual span), data points plotted above the diagonal 1∶1 line and in the shaded region represent improvement. In general, observers for the flanked letter training as a group showed improvement for all these measurements (all the bowtie symbols are in the shaded regions), even though these measurements were not used for training purpose. A paired t-test (t-statistics are given in File S1) confirmed that these improvements were significant, at the following p-values: (a) size limit, p = 0.035; (b) spacing limit, p = 0.019; (c) contrast threshold for single letters, p = 0.019; (d) size of visual span, p = 0.004.

We next examined whether the improvements observed as described above were specific to the training task, which consisted of visual stimuli with flankers, as learning to focus solely on the target letter in the presence of flankers could be a fundamentally different task from learning to identify a single letter presented on its own (see [28] for a review). To do so, we trained another group of six observers with amblyopia (four with strabismus and two without) using a letter training task that did not have flankers. This task, the “isolated letter training", targeted at improving an aspect of functional vision that is different from the spacing limit. Specifically, the isolated letter training task was designed to improve the contrast sensitivity for near-acuity letters, with an associated improvement in high-contrast visual acuity – i.e., the size limit. Because age may be an important determinant of the magnitude of improvement, we ensured that the average age of observers in this isolated letter training group was similar to that of the flanked letter training group (t-test: p = 0.60). The number of sessions and trials of training were identical to those of the flanked letter training group. We tracked the performance measurement during training, the contrast sensitivity (the reciprocal of contrast threshold, the minimum amount of contrast required) for identifying single near-acuity letters (see Materials and Methods for details). Training data for individual observers of this group are presented in the bottom row of Fig. 1. Similar to the flanked letter training, we quantified the improvements during the isolated letter training in three ways — (1) fitting a linear function to the training data of each observer and examining whether the slope of the linear function differs significantly from zero; (2) comparing the *expected* performance (based on the fitted linear function) between the first and the last block of trials; and (3) comparing the *empirical* performance between the first and the last block of trials. As shown in Fig. 1, using a linear function fit to the training data, we found that the slope for four of the six observers was statistically different from zero. This proportion of observers who did not show improvements was again, similar to those reported in previous studies [17], [32], [36]. When comparing the *expected* performance between the first and the last block of trials, the ratio between the two blocks averaged 0.80±0.23 (95% CI). This method yielded 95% confidence intervals that just marginally included a ratio of 1, implying that the improvement did not reach statistical significance at the 0.05 confidence level. When we computed the ratio in performance between the first and the last block of trials based on *empirical* data, the ratio averaged 0.69±0.16 (95% CI) and the 95% confidence intervals did not include the value of 1, meaning that the improvement for the group was significant at α = 0.05.

We also examined whether the improvement following training on the isolated letter task transferred to other visual tasks by comparing the same four measurements before and after training, as we did for the flanked letter training. As shown by the circular symbols in Fig. 4, except for one observer in panels b and d, the data for all other observers in this training group fall within the shaded regions. A paired t-test comparing the group-averaged data with the null effect confirmed that all these improvements were significant, at the following p-values: (a) size limit, p = 0.004; (b) spacing limit, p = 0.041; (c) contrast threshold for single letters, p<0.0001; (d) size of visual span, p = 0.038. Along with the results from the flanked letter training group, our results show that both training tasks were effective in inducing improvements on the letter size limit, letter spacing limit, letter contrast sensitivity and the size of visual span, regardless of whether the task was a trained or an untrained one.

### Generalization of the learning effect: dependency on the training task?

**Figure 5:**
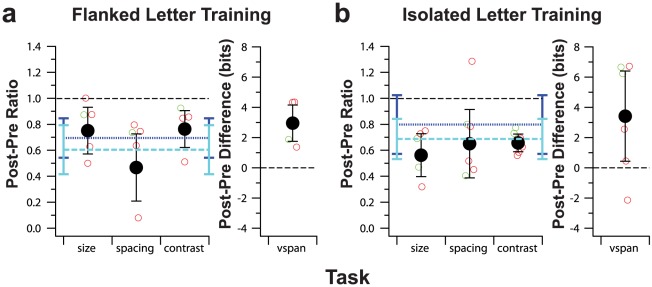
Post-pre ratios and difference comparisons for the four untrained visual tasks between the two training groups. **a.** Flanked letter training. **b.** Isolated letter training. Post-pre ratios were calculated for letter size limit (*size*), spacing limit (*spacing*) and contrast threshold for identifying single letters (*contrast*). Post-pre differences were calculated for the size of the visual span (*vspan*). Small unfilled symbols represent individual observers data with red representing strabismic amblyopes and green representing non-strabismic amblyopes. Black filled symbols represent the group-averaged value, with error bars representing the 95% confidence intervals. For comparison, the improvements due to training were included as blue lines (dark blue dotted line: ratio calculated based on the expected values for the first and the last block of trials derived from the linear functions fitted to the training data; light blue dashed line: ratio calculated based on the empirical performance for the first and the last block of trials). The ratio plotted for the training data was the pre-post ratio, instead of the post-pre ratio, as the performance accuracy was higher after training than before.

Our two training tasks were chosen on the basis that they targeted different limiting factors in amblyopic visual function. Specifically, our hypothesis was that the flanked letter training task would improve observers' ability to identify targets in clutter by reducing the effect of spatial crowding [29]. Thus, we expected that the spacing limit would benefit more from perceptual learning for the flanked letter training group than for the isolated letter training group. In contrast, based on the findings of Zhou et al [30] and Astle et al [18] showing that training on a contrast sensitivity measurement task improved letter acuity, we anticipated that the isolated letter training group might benefit more than the flanked letter training group on the size limit (visual acuity) and contrast threshold measurements for identifying single letters. To compare the effectiveness of the two training tasks on improving the various types of measurements, we computed the post-pre ratios for letter size limit, spacing limit and the contrast threshold for identifying single letters, for each observer. For the size of the visual span measurement, instead of computing the post-pre ratio, we computed the difference in bits of information transmitted (see Materials and Methods). Note that because the magnitude of the training effect depends on the pre-test value [2], we first confirmed that the pre-test values on these four measurements were not different between the two groups (t-test: p = 0.68 for size limit; p = 0.22 for spacing limit; p = 0.46 for contrast threshold for identifying single letters and p = 0.38 for size of the visual span). The post-pre ratios or differences for individual observers (small green or red symbols), as well as the group-averaged values (black filled symbols with ±95% confidence intervals), are plotted in Fig. 5a (flanked letter training) and 5b (isolated letter training). If the confidence intervals include a post-pre ratio of 1 for size, spacing and contrast threshold measurements, or a post-pre difference of 0 for visual span measurement, then we conclude that there was no statistically significant improvement in performance on the given task following training, at α = 0.05. For comparison, the improvements in performance for the trained task are also plotted in each panel (dark blue dotted line: ratio calculated based on the *expected* values derived from the linear function fitted to the training data; light blue dashed line: ratio calculated based on the *empirical* data). In general, the improvements were statistically significant for all four pre- and post-test measurements for the two training groups. For both training groups, the 95% confidence intervals for the size limit, spacing limit and contrast threshold for identifying single letters overlap with those of the training task (light or dark blue lines), implying a more or less complete transfer of learning to these untrained task. We are not able to draw the same conclusion for the visual span measurement simply because we compared the difference, instead of a ratio between the pre- and post-test measurement for visual span. Further, for each of the four measurements, the 95% confidence intervals between the two training groups overlap with each other, implying that the magnitude of improvements were similar between the two groups, consistent with the results of two-sample t-tests (size: p = 0.18; spacing: p = 0.93; contrast: p = 0.15; vspan: p = 0.88). In other words, the transfer of improvements to an untrained task did not depend on the training task.

Our initial expectation was that the flanked letter training would be more effective in reducing the spacing limit than the isolated letter training. However, Fig. 5 shows that the two groups seem to have benefited from a similar reduction in the spacing limit following their respective training. Presumably, learning to identify flanked letters leads to a reduction in the spacing limit, while improving letter acuity at the same time. However, to ask whether there was a specific reduction in crowding *per se*, we calculated a *crowding index*, defined as the ratio between the letter spacing limit and the letter size limit, for each observer. The post-pre ratio of this crowding index averaged 0.62±0.36 (95%CI) for the flanked letter training group, and 1.01±0.77 for the isolated letter training group. Although there were substantial individual differences, these values indicate that the flanked letter training led to a significant reduction in the crowding index, but not for the isolated letter training, implying that the flanked letter training might be more effective in reducing crowding *per se*.

## Discussion

Despite the conventional definition of amblyopia that is based only on a difference in visual acuity between the two eyes, individuals with amblyopia demonstrate visual deficits that affect a variety of visual tasks, including contrast sensitivity [33]–[35], contrast discrimination [37], relative position judgment [21], [38]–[40], contour integration [41]–[43], and second-order perception [31], [44], [45]. In addition, strabismic amblyopes are more susceptible to excessive spatial crowding [19]–[26]. If perceptual learning is to be used as an effective treatment for amblyopia, the improvements that follow the training should be generalizable to as many visual tasks as possible.

Here, we compared the effectiveness of two training tasks that were seemingly very different and were designed to improve different aspects of visual functions. The flanked letter training task has been shown to be effective in reducing the spacing limit in the normal periphery [29]. Because many spatial properties of strabismic amblyopia resemble those of the normal periphery, we predicted that the flanked letter training task would be effective in reducing the spacing limit, at least for strabismic amblyopes; however, we expected that its effectiveness on improving non-crowding related visual functions such as the (single) letter size limit might not be good. In contrast, the isolated letter training was designed to improve the contrast sensitivity for identifying single letters that were close to the acuity limit. Previously, Zhou et al [30] showed that visual acuity of a group of anisometropic amblyopes improved following training on a contrast sensitivity measurement using a single grating with a spatial frequency close to the high spatial-frequency cutoff (resolution limit). Astle et al [18] showed similar results using both single near acuity gratings and Landolt Cs. Therefore, we hypothesized that our isolated letter training would similarly lead to an improvement in letter acuity, but its effectiveness on improving visual functions such as reducing the spacing limit was unclear. The surprising finding of our study was that the two training tasks were more or less similarly effective in inducing improvements on the set of visual function measurements we performed, regardless of whether the task was a trained or an untrained one.

### Lack of specificity of learning?

The primary goal of this study was to determine whether perceptual learning could reduce crowding in adults with amblyopia. To this end, we first used a flanked letter training task that was specifically designed to reduce the spacing limit. Then to determine if the improvements were specific to the training task, *viz.*, the presence of flankers, we trained another group of amblyopic observers using a single letter training task that was supposed to improve the contrast sensitivity for near-acuity targets. Nevertheless we found that the improvements on a variety of visual tasks were similar between the two training groups. Therefore, it is reasonable to ask whether the similar magnitudes of improvements for the two training groups might be due to some generalized learning of observers learning how to do the task during the fairly extensive pre-testing. We do not think so because of the following reasons. First, prior to data collection (for the data reported in this paper), all observers were tested with 2–5 blocks (average = 3) of letter size threshold measurements (100 trials per block). The pre-test letter size threshold reported in this paper was obtained only after the threshold following each block appeared to have stabilized. As such, all observers had several hundred “practice trials" before actual data collection, which should be sufficient for any fast or general learning of performing the task to occur [11]. Second, as shown in Fig. 1, only observers AS, JS, SP and PT (four of the eleven observers) might have demonstrated a large improvement from the pre-test to the first block of training, which could be due to some general learning of how to perform the task. However, three other observers (GDW, BP and LA) actually showed a drop in performance from pre-test to the first block of training, while the rest of the observers (four of the eleven observers) showed similar performance between the pre-test measurement and their first block of training. These observations show that the presence of general learning from pre-test to the first training block, if any, is not a consistent finding across observers. To further quantify whether there was any evidence of significant improvements from pre-test to the first training block, we performed two analyses — (1) comparing the threshold estimate of the first training block with the pre-test value and (2) comparing the threshold estimate of the first half of the training block (first 50 trials) with that of the second half of the training block (last 50 trials). For both analyses and for both training groups, there was no evidence of significant differences in thresholds between the pre-test and the first training block, or between the first and the second halves of the first training block (see Table S1). Further, previous studies using a letter recognition training task that included a no-training control group invariably found no significant improvement for observers who only participated in the pre- and post-tests with no intervening training [18], [46], [47].

An alternative explanation for the apparent lack of specificity of learning is the common stimuli (i.e. letters) shared between the two tasks. Although the two training tasks were different, at the decision stage, observers were still required to match the perceptual input of the stimulus with a “template" in order to identify the target letter. For a variety of tasks such as position judgments [16], [48] and orientation discrimination [14], [15], improvements following perceptual learning have been attributed to a re-tuning of the perceptual template such that it is more capable of extracting the crucial information from the signal. Previously, we have shown that this mechanism for improvements also applies to learning to identify near-threshold low-contrast isolated letters [17], which was one of the two training tasks adopted in the current study. As for the flanked letter training task, a recent study suggests that the mechanism underlying the reduction of crowding following training is attributable to the perceptual window being more capable of adjusting its size to gather relevant input from the object of interest and its flankers [49]. Essentially, this also implies the capability of a perceptual or decision “template" to modify its characteristics to better tuned to the input stimulus. Based on this reasoning, it seems more likely to us that the apparent lack of specificity can be attributed to the fact that both training tasks, as well as all the pre- and post-test measurements, are related to a common stimulus (letters) and task, *viz.*, letter recognition. If perceptual learning serves to improve observers' ability to extract relevant information from the stimulus (letters in our case) and/or to improve the observer's decision “template", it seems reasonable to expect that performance on tasks related to letter identification would improve.

Our results are reminiscent of those of Polat et al [50], [51], Zhou et al [30], Liu et al [52] and Hussain et al [53]. By training a group of amblyopic observers to detect near-threshold Gabor stimuli with and without collinear high-contrast patches, Polat et al [50] showed that the improvements due to training were accompanied by higher sensitivity for the entire contrast sensitivity function, reduced crowding and higher letter acuity (see also [51]). Zhou et al [30] showed improvements on visual acuity and the contrast sensitivity function of a group of anisometropic amblyopes following training on a contrast detection task using a single grating of a spatial frequency close to the high spatial-frequency cutoff (resolution limit). Liu et al [52] also trained their amblyopic observers on a grating contrast detection task and found a small but significant improvement on contrast sensitivity, and single-letter or crowded-letter acuities. These studies imply that training on contrast detection of grating stimuli, with or without flankers, improves visual acuity and the contrast sensitivity function. Our training tasks, using letter stimuli instead of gratings, extend the findings of these earlier studies to show that the improvements on contrast sensitivity, crowding and letter acuity are not limited to using grating stimuli during training. A very recent study [53] trained amblyopes with letter targets and nearby flankers, and like us, showed that both flanked and unflanked acuity improved.

### Acuity improvement depends on training letter size?

Would any training task that utilizes letter identification be equally effective in improving the different visual function measurements as described here? We suspect not. In a previous study, we found that the improvement following perceptual learning on identifying near-threshold low-contrast single letters did not improve visual acuities [32], which seemed to contradict the finding from the current study. The difference in the finding might be attributable to the letter size used for training. In our previous study, the letter size was approximately 8× larger than the letter size limit, in sharp contrast to the 1.2× above the letter size limit used in the current study. This suggests that in order for learning to be generalizable to other conditions, the object size between the trained and untrained tasks need to be similar. Alternatively, perhaps the letter size is not a limiting factor, but instead, the improvement only generalizes from small to large objects. Huang et al [54] showed that practicing a contrast threshold measurement using a sine-wave grating with a spatial frequency close to the high spatial-frequency cut-off (resolution limit) led to an improvement in visual acuity (an untrained resolution task), with the effect spreading to spatial frequencies 4 octaves below the cut-off frequency. Therefore, the fact that we did not observe an improvement in visual acuity when the training letter size was 8× (∼3 octaves) larger than the resolution limit [32] could mean that the spread of learning is uni-directional such that the improvement only spread from small to large objects, but not in the opposite direction.

Note that this failure to find a generalized improvement to an acuity task following perceptual learning on a letter contrast sensitivity task simply adds to the list of studies that did not show generalized improvements on untrained tasks, even though the trained and untrained stimuli share similar attributes. These studies include one in which observers were trained to identify second-order (contrast-defined) single letters. Despite a substantial improvement in their ability to identify second-order letters following training, their ability to identify first-order (luminance-defined) single letters did not improve, suggesting a lack of transfer of learning [31].

### Caveats of the study

In this study, we did not include a no-training control group in our study design, so we cannot conclusively rule out the possibility that at least some observers may have shown some generalized learning from pre-test to the first block of training, although our analyses show that general learning is not a consistent finding across observers. However, we note that two observers in the isolated letter training group showed no improvement during the training (RE and SDW in Fig. 1), yet showed an improvement in the “size" and “contrast" tasks (Fig. 5b); and all but one observer showed an improvement in the “spacing" and “vspan" tasks. These improvements are not consistent with their training data, and could be explained by generalized learning. Alternatively, despite the absence of improvement on the trained task, these two observers may have learned something important during the extensive (10 kilotrials) training that is not evident in the performance on the trained task, but transferred to the sensitive pre-post training measures. Indeed, Liu et al [52] showed a similar effect in their previously patched group. These subjects showed no improvement on the trained grating acuity task; yet, they showed improvements on both isolated and crowded E acuities and stereoacuity.

Despite these caveats, we showed that our training tasks were effective in improving at least some aspects of letter recognition in adults with amblyopia. Clearly, if either of the two training tasks were to be used to treat amblyopia, a large-scale randomized clinical trial that includes a no-training control group would be necessary.

### Conclusions

We asked two groups of adults with long-standing amblyopia to perform different perceptual learning tasks: one group practiced the flanked letter training task [29], designed to reduce crowding in peripheral vision, while the other group practiced identification of small low contrast letters (isolated letter training task). Following training, observers in *both* groups demonstrated improved acuity and reduced crowding, higher sensitivity for identifying near-acuity letters and a larger visual span. We found that the two training tasks yielded similar magnitudes of improvements for the training tasks, as well as for a variety of untrained visual tasks. These improvements apparently did not depend on the type of amblyopia (strabismic or anisometropic).

## Materials and Methods

### Ethics Statement

The experimental procedures were approved by the Committee for the Protection of Human Subjects at the University of California, Berkeley. The research was conducted in accordance with principles expressed in the Declaration of Helsinki. All observers gave oral and written informed consent before the commencement of data collection.

### Participants

Eleven adult observers with amblyopia (eight with and three without strabismus), aged between 22 and 67 years, participated in this study. All were inexperienced with psychophysical experiments and naïve to the purpose of the experiment. The visual characteristics of these observers are summarized in Table 1. After the initial screening to establish that the observers were amblyopic, they were randomly assigned into the two training groups. Testing was performed using the amblyopic eye only, with the fellow non-amblyopic eye covered using a standard black eye-patch. All observers wore their best optical corrections for the viewing distance during testing.

### Stimulus Presentation

With the exception of visual-span profile measurement, stimuli were generated on a Macintosh G4 computer with software written in Matlab 5.2.2 (The MathWorks, MA) using the Psychophysics Toolbox extensions [55], [56] and were presented on a 17″ CRT monitor (Sony Trinitron CDP-G400) at a vertical refresh rate of 75 Hz. The background luminance of the display was 23 cd/m^2^.

For visual-span profile measurement, stimuli were generated on a PC (AMD Phenom processor based) with software written in Matlab 7 and were presented on a 21″ CRT monitor (Sony Trinitron GDM-F520) at a vertical refresh rate of 80 Hz. The background luminance of the display was 118 cd/m^2^.

### Stimuli

Stimuli were single letters or sequences of three letters (trigrams), randomly drawn (with replacement) from the 26 lowercase letters of the Times-Roman alphabet. Observers were asked to respond to the identity of the letters — single letters, the middle letter of each trigram, or all three letters in the visual span measurement — by typing their responses using a computer keyboard, following the disappearance of the stimulus on each trial. With the exception of visual span measurement (refer to the sub-section), the single letter, or the middle (target) letter of each trigram, was always presented at the center of the display. Two small dots, vertically straddling the target letter, were presented continuously on the monitor to act as fixation targets. Observers were asked to fixate the center between the two dots throughout testing.

### Pre-test

A set of baseline measurements was collected on each observer before training commenced. These measurements included (in the order they were measured): (1) letter size limit; (2) spacing limit; (3) contrast thresholds for identifying single letters; and (4) visual-span profile. The viewing distance was adjusted for each observer depending on the acuity measured using a standard Bailey-Lovie letter chart. Before the pre-test, each observer was tested with 2–5 blocks of trials (average = 3 blocks; 100 trials per block) using the same procedure as measuring the letter size limit. These served as “practice trials" to familiarize the observers with performing the letter identification task.

### Letter size limit

Five letter sizes (chosen such that observers' performance spanned a range from close to 0 to close to 100% correct) were each tested 20 times in a single block of trials. Observers responded to the identity of each single letter that was presented for a duration of 150 ms. Between 2 and 3 blocks of trials were tested for each observer. A cumulative Gaussian function was used to construct the psychometric function relating the proportion of correct letter responses to letter size. From the fitted cumulative Gaussian function, the letter size that corresponded to a proportion correct of 0.52 (equivalent to an identification accuracy of 0.5 after correction for guessing, chance level = 0.0384 [1/26]) on the psychometric function was defined as the letter size limit. Letter size was then set at 1.5× the letter size limit for subsequent testing during the pre-test. This letter size was chosen based on previous studies to avoid ceiling and floor effects in our measurements.

### Letter spacing limit

We measured the proportion correct of identifying the middle letter of trigrams for five center-to-center letter separations. Letter separations were specified as multiples of letter size in x-height, and ranged between 0.8× and 3× the x-height. Each letter separation was tested in a separate block of 20 trials. Identification accuracy was also measured for single letters. A cumulative Gaussian function was used to construct the psychometric function relating the proportion of correct letter responses to letter separation (Fig. 2). From the fitted cumulative Gaussian function, the letter separation (extrapolated if necessary) that corresponded to a proportion correct of 0.52 (chance level = 0.0384) on the psychometric function was defined as the letter spacing limit. This value was converted into the angular unit by multiplying it with the letter size in degrees.

### Contrast thresholds for identifying single or flanked letters

Single letters were presented at five levels of contrast in each block of trials (20 trials per contrast level). The levels of contrast were chosen such that observers' performance spanned a range from close to 0 to close to 100% correct. Observers responded to the identity of each single letter that was presented for a duration of 150 ms. To determine contrast threshold, a cumulative Gaussian function was used to construct the psychometric function relating the proportion of correct letter responses to the contrast of the target letter. From the fitted cumulative Gaussian function, the target letter contrast that corresponded to a proportion correct of 0.52 (chance level = 0.0384) on the psychometric function was defined as the contrast threshold.

### Visual-span profile

Visual-span profiles are plots of letter-recognition accuracy as a function of letter position left or right of the midline. It represents the amount of spatial information that the visual system could extract from the stimulus in a single eye fixation. Legge and colleagues [46], [57], [58] suggested that the size of the visual span, in bits of information transmitted, could impose a bottleneck on reading. Clearly, it would be advantageous to have a larger visual span. In this study, we adopted the method outlined in Legge et al [57] to measure the visual-span profile. In brief, sequences of three letters (trigrams) were presented along a horizontal meridian centering on the observer's fixation (letter position 0). The position of each trigram was indexed by the middle letter, and extended to 7 letter positions to the right (+) and left (−) of fixation. Observers were asked to identify all three letters in each trigram, from left to right, guessing if necessary. Ten trigrams were tested at each letter position in a random order, with a total of 150 trials tested in each block. This resulted in an accumulated number of 30 letter presentations at each letter position from −6 to +6 (10 presentations with the letter being the left letter of a trigram, 10 the middle letter and 10 the right letter). We then constructed a plot with proportion correct of responses as a function of letter position and fit the data using a split-Gaussian function (Fig. 3). Using the fitted function, we converted the performance accuracy at each letter position into bits of information transmitted using the following empirically derived equation [57], taken into account the confusion matrices for single letter identification [59]:
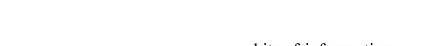
Information transmitted at a given letter slot ranged from 0 bit (for chance accuracy of 0.0384) to approximately 4.7 bits (for perfect identification). This conversion allowed us to quantify the size of the visual-span profile by summing the bits of information transmitted across all letter slots of the visual-span profile, akin to integrating the area under the visual-span profile with a scale change to express the result as bits of information.

### Flanked letter training

Stimuli for the flanked letter training consisted of trigrams, with letters presented at 100% contrast, for a duration of 150 ms. Letter size was set at 1.5× the pre-test letter size limit. The center-to-center separation between adjacent letters was 0.8× the letter size (x-height), as in Chung [29]. Observers identified the middle letter of each trigram. Training consisted of 10 sessions, with 10 blocks of trials (100 trials per block) tested per session. The 10 kilotrials were more than the 6000 trials used in Chung [29], and also more than most of the studies on perceptual learning in amblyopia, to ensure that we got a sizeable learning effect. On average, observers completed the 10 training sessions in two weeks. One observer, JHS, however, showed no improvement after 10 sessions, therefore we continued with the training for an additional 16 sessions (for a total of 26 sessions).

### Isolated letter training

Stimuli for the isolated letter training consisted of single letters presented at five levels of contrast, for a duration of 150 ms. For this training, we chose a letter size slightly smaller than the one used for the flanked letter training task, 1.2× the pre-test letter size limit, because of the following reasons. Zhou et al [30] showed that training using a single spatial-frequency grating closed to the high spatial-frequency cut-off of the contrast sensitivity function subsequently improved the visual acuity of a group of observers with anisometropic amblyopia. However, in a previous study of ours [32], we trained a group of amblyopic observers (strabismic and non-strabismic) on identifying low-contrast letters, and failed to observe an improvement in visual acuity even though most of the observers improved on the training task. We suspected that this failure to observe a transferred improvement on visual acuity following training on identifying low-contrast letters was related to the relatively large letter size used in our previous study. Therefore, in this study, we chose a letter size much closer to the acuity limit. Similar to the flanked letter training, observers completed a total of 10 sessions, with 10 blocks of trials tested per session.

### Post-test

The post-test, identical to the pre-test, except that the order of testing of the different measurements was conducted in the reverse order, was performed (within one to three days) after the last training session.

## Supporting Information

File S1 List of t-statistics and p-values pertinent to Figures 4 and 5.
(DOC)

Table S1 Comparisons of pre-test measurement with the first training block, and the first half vs. the second half of the first training block, to determine if there was any significant improvement between pre-test and the first block of training, or during the initial training. For the flanked letter training group, because a change in performance from 0.1 to 0.2, or 0.6 to 0.7 are not the same, we first converted each proportion-correct score into *z*-score and then calculated the difference in *z*-score values. We then compared the z-score values against a value of 0 (signifying no improvement) using *t*-test to determine if there was any improvement between the measurements. For the isolated letter training group, because the thresholds were in contrast unit, we calculated the ratio of the contrast thresholds and compared the ratios against a value of 1 (no difference in thresholds between the two measurements).
(DOC)
