# Supplementary material for: Learning to Identify Near-Acuity Letters, either with or without Flankers, Results in Improved Letter Size and Spacing Limits in Adults with Amblyopia
Source: PLoS One. 2012 Apr 30;7(4):e35829. doi: 10.1371/journal.pone.0035829 (PMC3340394; doi:10.1371/journal.pone.0035829)
Supplement: File S1 — List of t-statistics and p-values pertinent to Figures 4 and 5. (DOC) [file pone.0035829.s001.doc]

**File S1.**

Flanked Letter Training Group (Figure 4: bowtie symbols only)

Post-pre ratio (PPR) of size limit (panel 4a):

mean±95%CI = 0.78±0.18,

t-test to compare with a null PPR of 1: t (df=4) = 3.13, one-tailed p = 0.035

Post-pre ratio (PPR) of spacing limit (panel 4b):

mean±95%CI = 0.60±0.26,

t-test to compare with a null PPR of 1: t (df=4) = 3.80, one-tailed p = 0.019

Post-pre ratio (PPR) of contrast (panel 4c):

mean±95%CI = 0.78±0.14,

t-test to compare with a null PPR of 1: t (df=4) = 3.82, one-tailed p = 0.019

Post-pre difference of vspan (panel 4d):

mean±95%CI = 2.95±1.20,

t-test to compare with a null value of 0: t (df=4) = 5.83, one-tailed p = 0.004

Isolated Letter Training Group (Figure 4: circular symbols only)

Post-pre ratio (PPR) of size limit (panel 4a):

mean±95%CI = 0.59±0.17,

t-test to compare with a null PPR of 1: t (df=4) = 5.89, one-tailed p = 0.004

(note that we did not have the post-test size measurement for SDW)

Post-pre ratio (PPR) of spacing limit (panel 4b):

mean±95%CI = 0.71±0.26,

t-test to compare with a null PPR of 1: t (df=5) = 2.75, one-tailed p = 0.041

Post-pre ratio (PPR) of contrast (panel 4c):

mean±95%CI = 0.66±0.07,

t-test to compare with a null PPR of 1: t (df=5) = 11.3, one-tailed p < 0.0001

Post-pre difference of vspan (panel 4d):

mean±95%CI = 3.41±2.99,

t-test to compare with a null value of 0: t (df=5) = 2.81, one-tailed p = 0.038
