# Supplementary material for: Learning to Identify Near-Acuity Letters, either with or without Flankers, Results in Improved Letter Size and Spacing Limits in Adults with Amblyopia
Source: PLoS One. 2012 Apr 30;7(4):e35829. doi: 10.1371/journal.pone.0035829 (PMC3340394; doi:10.1371/journal.pone.0035829)
Supplement: Table S1 — Comparisons of pre-test measurement with the first training block, and the first half vs. the second half of the first training block, to determine if there was any significant improvement between pre-test and the first block of training, or during the initial training. For the flanked letter training group, because a change in performance from 0.1 to 0.2, or 0.6 to 0.7 are not the same, we first converted each proportion-correct score into z-score and then calculated the difference in z-score values. We then compared the z-score values against a value of 0 (signifying no improvement) using t-test to determine if there was any improvement between the measurements. For the isolated letter training group, because the thresholds were in contrast unit, we calculated the ratio of the contrast thresholds and compared the ratios against a value of 1 (no difference in thresholds between the two measurements). (DOC) [file pone.0035829.s002.doc]

**Table S1.**

| **Flanked letter training group** | | | |  |  |  |  |
| --- | --- | --- | --- | --- | --- | --- | --- |
| Observer | pretest | 1st blk | z-score diff |  | 1st half of 1st blk | 2nd half of 1st blk | z-score diff |
| GDW | 0.325 | 0.25 | -0.221 |  | 0.18 | 0.1 | -0.366 |
| AS | 0.1 | 0.4 | 1.028 |  | 0.42 | 0.36 | -0.157 |
| BP | 0.35 | 0.05 | -1.260 |  | 0.1 | 0.14 | 0.201 |
| JHS | 0.1 | 0.15 | 0.245 |  | 0.08 | 0.22 | 0.633 |
| JS | 0.2 | 0.45 | 0.716 |  | 0.38 | 0.42 | 0.104 |
|  |  |  |  |  |  |  |  |
| AVE |  |  | 0.102 |  |  |  | 0.083 |
| p-value (ttest: different from 0?) | | | 0.812 |  |  |  | 0.650 |
|  |  |  |  |  |  |  |  |
| **Isolated letter training group** | | | |  |  |  |  |
| Observer | pretest | 1st blk | ratio |  | 1st half of 1st blk | 2nd half of 1st blk | ratio |
| SP | 0.164 | 0.100 | 0.610 |  | 0.097 | 0.104 | 1.073 |
| SDW | 0.137 | 0.156 | 1.134 |  | 0.160 | 0.153 | 0.959 |
| PT | 0.640 | 0.360 | 0.562 |  | 0.420 | 0.308 | 0.734 |
| RE | 0.360 | 0.427 | 1.186 |  | 0.356 | 0.536 | 1.508 |
| JL | 0.194 | 0.432 | 2.229 |  | 0.513 | 0.343 | 0.669 |
| LA | 0.263 | 0.422 | 1.604 |  | 0.479 | 0.373 | 0.779 |
|  |  |  |  |  |  |  |  |
| AVE |  |  | 1.221 |  |  |  | 0.953 |
| p-value (ttest: different from 1?) | | | 0.429 |  |  |  | 0.728 |
